# Pollution Characteristics and Risk Assessment of Heavy Metals in the Sediments of the Inflow Rivers of Dianchi Lake, China

**DOI:** 10.3390/toxics12050322

**Published:** 2024-04-29

**Authors:** Liwei He, Guangye Chen, Xinze Wang, Jian Shen, Hongjiao Zhang, Yuanyuan Lin, Yang Shen, Feiyan Lang, Chenglei Gong

**Affiliations:** 1 Yunnan Dali Research Institute of Shanghai Jiao Tong University, Dali 671000, China; heliwei2010@yeah.net; 2 School of Chemistry and Chemical Engineering, Kunming University, Kunming 650214, China; 3 National Observation and Research Station of Erhai Lake Ecosystem in Yunnan, Dali 671000, China

**Keywords:** heavy metal pollution, chemical speciation, risk assessment, source identification

## Abstract

To explore the contamination status and identify the source of the heavy metals in the sediments in the major inflow rivers of Dianchi Lake in China, sediment samples were collected and analyzed. Specifically, the distribution, source, water quality, and health risk assessment of the heavy metals were analyzed using correlation analysis (CA), principal component analysis (PCA), the heavy metal contamination factor (*C_f_*), the pollution load index (*PLI*), and the potential ecological risk index (*PERI*). Additionally, the chemical fractions were analyzed for mobility characteristics. The results indicate that the average concentration of the heavy metals in the sediment ranked in the descending order of Zn > Cr > Cu > Pb > As > Ni > Cd > Hg, and most of the elements existed in less-mobile forms. The *C_f_*was in the order of Hg > Zn > Cd > As > Pb > Cr > Ni; the accumulation of Hg, Zn, Cd, and As was obvious. Although the spatial variability of the heavy metal contents was pronounced, the synthetical evaluation index of the *PLI* and *PERI* both reached a high pollution level. The PCA and CA results indicate that industrial, transportation, and agricultural emissions were the dominant factors causing heavy metal pollution. These results provide important data for improving water resource management efficiency and heavy metal pollution prevention in Dianchi Lake.

## 1. Introduction

With the rapid development of urbanization and industry, large amounts of pollutants, including organic matter pollutants, nutrients, and particulate matter, are being released into the water system through various pathways [1,2]. Heavy metals have gained attention for their characteristics of strong toxicity, bioaccumulation, and nonbiodegradability, thereby posing a threat to ecology and the food chain, and consequently to human health [3,4,5]. In current aquatic systems, the potential risk of heavy metal pollution has become a major global environmental challenge and has elicited significant concern from governments and the public [6,7]. Heavy metal contamination has been reported in the rivers and lakes of many countries and regions in the world [8,9,10]. For instance, heavy metal concentration has increased greatly in five continents since the 1900s [11]. The human activities that release heavy metals into the environment mainly include industrial discharge, agriculture, and mining [12,13]. Many studies have shown that sediment is an important reservoir of various substances in a water body, and it usually contains much higher pollutant contents than those in the water. This is because of the strong accumulation effect of most pollutants, which means that the sediment serves as a large sink for heavy metals [14,15]. Metals accumulate through physical adsorption, chemical precipitation, and biodegradation [16,17]. However, when the external conditions change, the metals in the sediment can be released into the water body, which can threaten aquatic ecosystems and have significant impacts on human welfare [18,19].

Consequently, risk assessment of heavy metals is important for water safety and watershed management. In traditional studies of heavy metal pollution, the spatial distribution of different heavy metal pollutants is often used to assess the impact of anthropogenic and natural factors on aquatic ecosystems [20]. However, some studies have shown that heavy metal concentration data alone do not accurately reflect the sediment contamination status, environmental quality, and possible adverse effects on benthic organisms [21]. The transportation of heavy metals between the sediment and the overlay water is an important factor for the analysis of the metal pollution status, and heavy metal concentration in the sediment is regarded as an indicator for monitoring the contamination in aquatic ecosystems [13,22]. Furthermore, many studies have shown that it is possible to determine the source, distribution, extent, and possible hazards of metal contamination by analyzing the sediments in the surface water [23,24]. 

Several methods have been used to analyze the pollution level and evaluate the risk of heavy metals in the sediment of aquatic systems. The methods for calculating the risk of individual heavy metals usually include an enrichment factor, contamination factor (*C_f_*), pollution index (*PLI*), and geo-accumulation index, and the total content and background value or sediment quality guideline value of the specific elements are used as a guideline [25,26]. Methods for evaluating multiple heavy metal risks in the sediment have also been proposed, such as the *PLI*, the mean probable effect concentration quotient, and the potential ecological risk index (*PERI*) [23,27,28]. Most research indicates that the combined use of these methods is more reliable [29]. The chemical fractions of the heavy metals in sediments are also considered reliable for the determination of heavy metal toxicity [30]. This is because the available form is highly active and has a much closer relationship to anthropogenic sources [31]. 

Dianchi Lake is the sixth largest freshwater lake in China and is a typical plateau lake. It is located in the middle of the Yunnan–Guizhou Plateau at an elevation of 1900 m [32]. It is downstream of Kunming and provides water for industry, agriculture, and residential use around the lake [33]. It is also the lowest concave area of Kunming Basin, and, thus, it has become the “sewage bucket” of Kunming. Due to the increase in human activities and urbanization, its water quality has decreased and it suffers from serious water pollution. The lake has been included in the National Key River Basin Planning on four consecutive occasions. Heavy metal pollutants like copper (Cu), cadmium (Cd), zinc (Zn), lead (Pb), and arsenic (As) have been detected in Dianchi Lake [34,35]. Although the comprehensive treatment of this lake is one of the key tasks of the government, there is urban sewer runoff and agricultural nonpoint source pollution from Kunming that drains into it [36]. As most previous studies have focused on the water body, research on the sediment, especially that of the inflow river, is insufficient. Therefore, there is a need to assess the impact of human activities on the water environment in terms of sediments. 

The estuaries of the 12 inflow rivers of Dianchi Lake were selected as the focus of this study, and the heavy metal concentrations, nutrient concentrations, and pH in the sediment and overlay waters were investigated. The objectives of this study were to (1) investigate the distribution characteristics of the heavy metals in the sediment and overlay water; (2) evaluate the contamination level and potential health risk of heavy metal accumulation using multivariate indexes; and (3) analyze the sources of the heavy metals in the study area. The findings of this study complement the existing data on the heavy metal contents in the sediment in Dianchi Lake and contribute to the pollution management strategies for reducing future heavy metal pollution risks.

## 2. Materials and Methods

### 2.1. Study Site Description and Sampling

**Figure 1:**
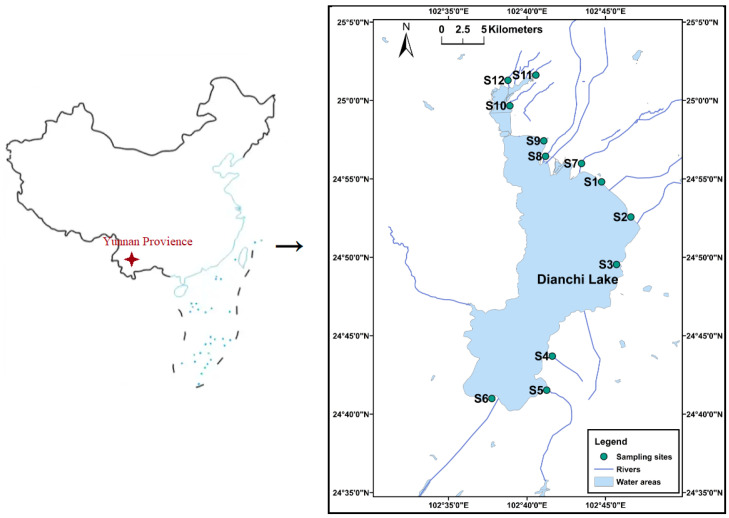
Sampling site distribution.

Dianchi Lake is a large, eutrophic shallow lake in Kunming (24°23′–26°22′ N, 102°10′–103°40′ E). Its water area is 2920 km^2^ in extent, with an average water depth of 4.4 m, and the shallow lake body is divided into two parts; the northern inner lake is called Cao Hai, and the much larger southern outer lake is called Waihai [37]. There are at least 17 rivers and 20 springs flowing into the lake, and they enter it from three directions: east, south, and north. The 12 main rivers with large runoff and serious pollution were selected for sample collection. The sediments and upper overlay water at the same sample point were collected in October 2021 and February 2022, at the end of the wet season and the dry season, respectively. Three or more surface sediment samples (0–20 cm) were collected at each sampling point using a sediment sampler. The sampling points and study area are illustrated in Figure 1. 

The water quality indicators, including temperature, pH, dissolved oxygen, and conductivity, were measured at each site. An additional 500 mL water sample was collected and acidified at each site for the analysis of the heavy metal concentration in the water in the laboratory.

The collected sediments were air-dried at room temperature. Then, the sand, shellfish, and plant residues were removed. After that, the dry and clean samples were ground with an agate grinder and sieved to pass through a 200-mesh screen (~75 μm) and stored in a polyethylene plastic bag for future use.

### 2.2. Laboratory Analyses

#### 2.2.1. Water Quality Index Analysis

The total nitrogen (TN) was measured with an ultraviolet spectrophotometer (UV1900, Phenix, Shanghai, China). The total phosphorus (TP) was measured with an Auto Analyzer 3 (Seal Analytical, Shanghai, China). The permanganate index (COD_Mn_) was determined according to the PRC national standard GB 11892-1989 [38]. 

The chlorophyll a (Chl-a) was detected according to the PRC national standard HJ 897-2017 [39]. An ultracentrifuge was used for the centrifugation of the extracts, and a horizontal rotator shaker was used for the extraction. All the glass containers were soaked in 50% HNO_3_ (*v*/*v*) and rinsed with deionized water. The extracts from the samples were stored in PTFE tubes at 4 °C before analysis. 

#### 2.2.2. Eutrophication Index Calculation

The eutrophication degree of the water body was evaluated using the comprehensive nutrient state index (*TLI_∑_*), and the calculation formula is as follows:

(1) $${TLI}_{\sum }=\sum_{j=1}^{m}W_{j}\times TLI$$

where *W_j_* is the relative weight of the nutritional status index of the *j*th parameter, and *m* is the number of evaluated parameters, including Chl-a, TP, TN, SD, and COD_Mn_. Using Chl-a as the basic reference parameter, the normalized relevant weight calculation formula of the parameter is as follows:

(2) $$W_{j}=r_{ij}^{2}/\sum_{j=1}^{n}r_{ij}^{2}$$

where $r_{ij}^{2}$ is the correlation coefficient of the *j*th parameter and basic standard indexes (Chl-a). A series of continuous numbers ranging from 0 to 100 were used to grade the nutrient status of the lakes. The higher the index value was, the heavier the nutrient level was. The values of $r_{ij}$ and $r_{ij}^{2}$, the calculation formula, and the grading values at the different levels are provided in the Supporting Information (Tables S1 and S2).

#### 2.2.3. Digestion of Sediments 

The total concentrations of Cu, Zn, As, Cd, Pb, nickel (Ni), and mercury (Hg) in the sediment water were measured: 0.50 g (accurate to 0.0001 g) of ground sediment sample was digested with 6 mL of nitric acid (HNO_3_), 2 mL of hydrofluoric acid (HF), 3 mL of hydrochloric acid (HCl), and 1 mL of hydrogen peroxide (H_2_O_2_) in a Teflon beaker using a microwave digestion system (WX-8000) [40]. The digested solutions were filtered through a 0.45 μm cellulose acetate membrane to remove the precipitates before instrumental analysis. 

The standard reference materials (TMQCO251) and blanks were analyzed at regular intervals for quality control and method validation, and the blanks were analyzed with each round of digestion to monitor the contamination and background metal concentration in the reagents and digestion process. The recovery rate of the detected metals ranged between 90% and 110% for the sediment reference material, and the relative error was less than 10%, which indicated that the quality of the sample digestion was within the acceptable range.

#### 2.2.4. Sequential Extraction Process of the Sediment

A modified Tessier extraction method was conducted to sequentially extract the chemical forms of the metals in the sediments, and the main procedure is briefly described below [41]. 

Extractable fraction (F1): A 1.0000 g (accurate to less than 0.0003 g) soil sample and 24 mL of 0.5 M magnesium chloride (MgCl_2_·6H_2_O) were added into a 50 mL centrifuge tube. The samples were oscillated at 200 r/min for 20 min at room temperature and centrifuged for 20 min at 4000 r/min. The supernatant solution was then collected, and the volume was fixed and prepared for analysis. 

Carbonate fraction (F2): The residue after the previous treatment was extracted with 24 mL of 1 M sodium acetate at room temperature. Before the extraction, the pH was adjusted to 5.0 using acetic acid (HAc), it was shaken at 200 r/min for 5 h, and then centrifugation was conducted at 4000 r/min for 20 min. The supernatant was removed and filtered, and then it was fixed for analysis. 

Fe/Mn oxide fraction (F3): The sediments from the previous step were added to a 25% (*v*/*v*) HAc solution and 20 mL of 0.04 M hydroxylamine hydrochloride (NH_2_OH·HCl). Then, the samples were extracted at 96 ± 3 °C for 6 h, centrifugation was performed for 20 min at 4000 r/min, and the supernatant was removed and stored for the metal concentration analysis. 

Organic matter fraction (F4): Initially, 3 mL of 0.02 M nitric acid (HNO_3_) and 5 mL of 30% (*v*/*v*) H_2_O_2_ were added to the sediments from the previous step, the pH was adjusted to 2 with HNO_3_, the mixture was heated to 85 ± 2 °C for 2 h, and oscillation was conducted several times during the heating. Then, 5 mL of 30% H_2_O_2_ was added to adjust the pH to 2, the mixture was heated to 85 ± 2 °C for 3 h, and it was oscillated intermittently. After cooling, 5 mL of 3.2 mol/L ammonium acetate was added, it was diluted to 20 mL with 20% HNO_3_, and it was shaken for 30 min. The supernatant was extracted by centrifugation for 10 min at 4000 r/min, and it was stored for later analysis. 

Residual fraction (F5): The residue from the previous step was digested and analyzed using the HNO_3_-HF-HClO_4_ digestion method. The supernatant solution was then collected and stored for the metal concentration analysis.

#### 2.2.5. Element Determination

The concentrations of Cu, Zn, Pb, Ni, and As were measured using X-ray fluorescence spectrometry (S8 Tiger) according to the published PRC national analytical standard HJ 780-2015 [42], the Cd concentration was measured using flame atomic absorption spectrometry (ICE-3500) according to the PRC national standard GB/T 17141-1997 [43], and the Hg concentration was measured using atomic fluorescence spectrometry (AFS-230E) according to the PRC national standard HJ 680-2013 [44].

### 2.3. Method for Pollution Risk Evaluation

#### 2.3.1. Contamination Factor

The contamination factor ($C_{f}$) is a simple and powerful measure for identifying the contamination of toxic metals based on the ratio of the estimated values of the metals to their background values [45], which is expressed in Equation (3):

(3) $$C_{f}^{i}={\;C}_{i}/B_{i}$$

where $C_{f}^{i}$ is the contamination factor of metal *i*, and $C_{i}$ and $B_{i}$ are the determined value and background value of the heavy metal *i*, respectively. 

#### 2.3.2. Pollution Load Index

The *PLI* is calculated by using the geometric mean of *n* $C_{f}^{i}$ and integrating all the determined metals [46]. It is expressed in Equation (4):

(4) $$PLI={(C_{f}^{1}\times {\;C}_{f}^{2}\times \ldots \ldots \times \;C_{f}^{n})}^{1/n}$$

#### 2.3.3. Potential Ecological Risk Index

The potential ecological risk index (*PERI*) is a measure for analyzing the cumulative pollution level that takes into full consideration the migration, transformation, and toxicity of heavy metals [45]. The index is shown in Equations (5) and (6):

(5) $$E_{r}^{i}={\;T}_{r}^{i}\times C_{f}^{i}$$

(6) $$PERI=\sum_{i=1}^{n}E_{r}^{i}=\sum_{i=1}^{n}T_{r}^{i}\times C_{f}^{i}=\sum_{i=1}^{n}T_{r}^{i}\times \frac{C_{i}}{B_{i}}$$

where $E_{r}^{i}$ is the ecological risk index of element *i* in the sediment (*i* ranges from 1 to n heavy metal elements as assessed in this study), and $T_{r}^{i}$ is the toxicity coefficient of the heavy metal *i*, which is predefined according to the main hazard path of the eight elements ($T_{r}^{i}$; Hg = 40, Cd = 30, As = 10, Pb = 5, Cu = 5, Cr = 2, Ni = 2, and Zn = 1) [45].

### 2.4. Method for Source Identification

The statistical analysis was conducted using SPSS software (23.0). The estimation of the potential sources of the metals in the sediments of Lake Dianchi was conducted using correlation analysis (CA) and principal component analysis (PCA), which have been widely used for source apportionment and to characterize the interactions of heavy metals and the physicochemical indexes of polluted areas [46]. Based on the principle of dimensionality reduction, and minimizing the loss of original information, several interrelated variables were removed. 

## 3. Results and Discussion

### 3.1. Water Quality and Chemical Properties 

The analyzed water environmental quality index of the inflow rivers of Dianchi Lake in the survey is displayed in Table 1. The heavy metal concentrations in the inflow rivers fell below the detection limit except for As. The average value of the *TLI*_∑_ index of the 12 sample sites indicated the mesotrophic level (43.3), and the *TLI_∑_* index value was higher in the wet season than in the dry season. For the water quality of the 12 sample points, S1 to S8 belonged to the class III water quality standard, while S9 to S12 belonged to the class Ⅳ water quality standard according to PRC national standard GB 3838-2002 [47]. The water quality of the inflow rivers in the north of Dianchi Lake was worse than that in the south, which was in accordance with the population density distribution. 

The coefficient of variation (CV) is usually used to indicate the strength of the interference due to human activities [48], and it was classified into three grades: CV < 15% indicated low variability, 15% < CV < 36% indicated moderate variability, and CV > 36% indicated high variability [49]. The CV of the parameters showed that the degree of eutrophication was relatively moderate, and the water quality indexes, including Chl-a, COD_Mn_, TN, TP, and As, were highly variable. Thus, it could be concluded that the decrease in the water quality may have originated from anthropogenic activities, and the CV value of Chl-a was the highest, which indicated that the algae biomass had the strongest correlation with anthropogenic activities. These results showed that the inflow rivers were one of the sources of eutrophication in the major water body of Dianchi Lake. Table 2 provides a comparison of the water quality index in the wet season and dry season; the Hg, Pb, As, Cd, Cu, Zn, Ni, and Cr content ranges were significantly different between seasons, and the variance analysis showed that the COD_Mn_, As, and TN were also significantly different between seasons. The Chl-a, TP, and *TLI*_∑_ were not significantly different. Thus, following the source apportionment analysis, the *TLI*_∑_ indexes in the different seasons were analyzed as one group. 

### 3.2. Heavy Metal Distribution and Chemical Properties 

#### 3.2.1. Content of the Total Heavy Metals 

The distribution of the heavy metals in the 12 study sites was spatially heterogeneous. As displayed in Table 3, the Hg, Pb, As, Cd, Cu, Zn, Ni, and Cr content ranges were 0.06–3.29 mg/kg, 25.8–122 mg/kg, 6.7–96.6 mg/kg, 0–11.1 mg/kg, 25.8–227 mg/kg, 61.7–689 mg/kg, 0–48.9 mg/kg, and 61–220 mg/kg, respectively. The average concentration of the heavy metals in the sediment of Dianchi Lake ranked in descending order were Zn > Cr > Cu > Pb > As > Ni > Cd > Hg, and the times to the background values ranked from high to low was Hg (11.83) > Cd (4.00) = As (4.00) > Zn (3.23) > Pb (1.84) > Cu (1.68) > Cr (1.23) > Ni (0.14). 

The background values for the evaluation of the heavy metals based on the total content were derived from the results of the deep sediment samples of the National Multi-Purpose Regional Geochemical Survey Project [50]. Among the detected heavy metals, except for Hg, the value of the other seven elements all exceeded the background values, which indicated that there was heavy metal contamination in the sediment. The results of the CV indicated that the variability of the heavy metals ranked in descending order was Ni > Cd > Hg > As > Zn > Cu > Pb > Cr. The element Cr had moderate variability, and the other elements were all highly variable, which suggests that the heavy metal pollution in the sediment was largely caused by human activities. The results of the heavy metal content in the inflow river sediment of Dianchi Lake in the wet and dry seasons are summarized and compared in Table 4. A one-way analysis of variance was performed to test the differences in the heavy metal concentration during dry and wet seasons, and the P-values were all above 0.1. This indicated that the differences were not significant and that the seasonal impact was not obvious. 

#### 3.2.2. Chemical Speciation of the Heavy Metals 

**Figure 2:**
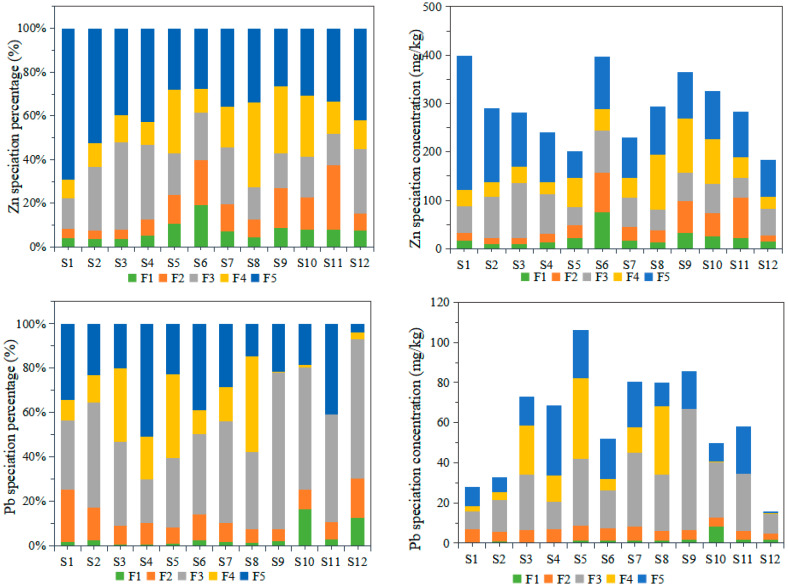

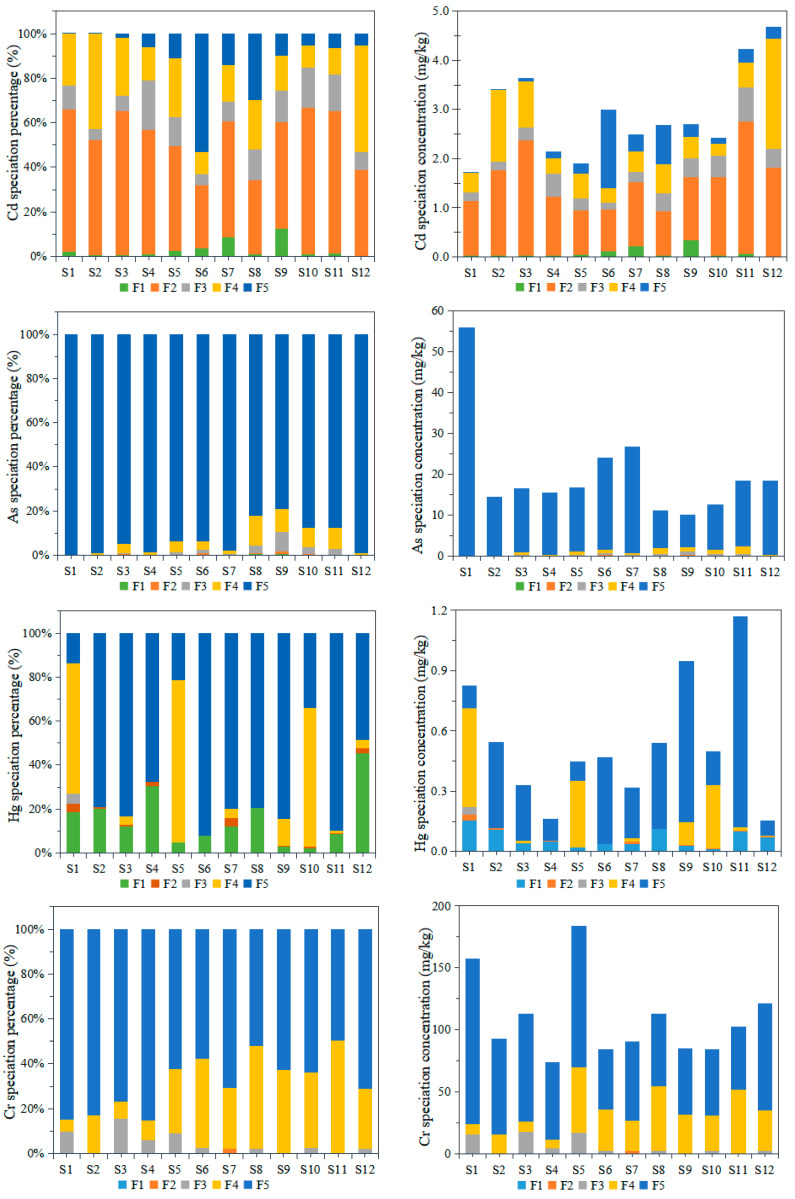
Chemical form distribution for different heavy metals.

Although total concentration is commonly used for heavy metal contamination assessment, it was shown to be inaccurate in evaluating the effects of the pollutants on the ecosystem since the mobility, bioaccumulation, and toxicity of heavy metals vary with different chemical forms [51,52]. In this study, chemical speciation analysis was conducted using a sequential extraction method. Five chemical forms, namely, the extractable fraction, carbonates, Fe/Mn oxides, organic matter, and residual fraction, were measured for seven of the heavy metals (Figure 2). The concentration of the different chemical forms of Ni was not discussed because it was hardly detected in most of the samples (below the detection limit). The results illustrated that there was an apparent discrepancy in the proportion of the different chemical species for the different heavy metals in each sample site.

Previous research has indicated that the most mobile and bioavailable form of sediment is the extractable fraction, and the higher the percentage of this fraction, the higher the mobility and the more likely it is to be of anthropogenic origin [53]. The carbonate fraction can be released under mildly acidic conditions and can also be regarded as bioavailable. The Fe/Mn oxide fraction and organic matter form are usually considered to be not bioavailable, but they can be released as active forms when the sediment environmental conditions change, such as the sediment pH, organic matter content, carbonate content, oxidation-reduction potential, and iron and manganese oxide content [54]. The residual fraction is the most stable form in the sediment, which is due to the crystal lattice structure of the primary and secondary minerals [27]. Hence, the stability of the different chemical forms in increasing order was extractable < carbonate-bound < Fe/Mn oxide-bound < organic matter-bound < residual fraction.

In this study, Cu mainly existed in residual and organic matter-bound fractions, and the concentration of the two factions was over 80% (84.81–100%) in all the sample sites. The proportion of the residual form was comparably higher at most of the sites, and the average concentration percentage reached 67.39% at the twelve sites, which indicated that Cu speciation in the sediment was stable. The average percentage of the different forms of Zn in the sediment in decreasing order was F5 (38.52%) > F3 (23.08%) > F4 (18.86%) > F2 (12.03%) > F1 (7.51%). Additionally, Zn mainly existed in the residual, Fe/Mn oxide-bound, and organic matter-bound fractions, the residual form was the highest proportion, and the average proportion of the first four forms amounted to 92.49% of the total concentration. Thus, the results indicated that the Zn in the sediment was comparatively stable.

The average proportion of the Pb fraction forms ordered from high to low was F3 (43.43%) > F5 (26.48%) > F4 (15.42%) > F2 (10.77%) > F1 (3.89%). Furthermore, Pb mainly existed in the Fe/Mn oxide-bound fraction. The proportion of Cd fractions from high to low was F2 (51.11%) > F4 (22.28%) > F5 (11.92%) > F3 (11.82%) > F1 (2.87%). The results indicated that Cd mainly existed in the carbonate fraction, and the organic matter-bound form was the second most common. These two forms contributed 73.39% to the total concentration. Thus, the Cd in the sediment was comparatively mobile and more bioavailable. For As, the average proportion of the residual form was over 90% of the total concentration (92.74%). Thus, the mobility and bioavailability of As in the sediment was extremely low. The proportion of Hg fractions from high to low was F5 > F4 > F1> F2 > F3, and the residual form made up over 60% of the total concentration of Hg (64.42%). The second most abundant fraction was the organic matter-bound fraction, and the Fe/Mn oxide-bound form was the lowest (0.37%). The distribution of the chemical fraction of Cr was similar to that of Hg, and the average proportions of the residual and organic matter-bound fractions were 68.30% and 27.39%, respectively. 

Overall, the results of the different sites showed that the mobility and chemical fraction characteristics varied for the different heavy metal elements. However, the bioavailability of the heavy metals, excluding Ni, from high to low was Cd (53.98%) > Zn (19.54%) > Hg (16.67%) > Pb (14.67%) > As (0.48%) > Cr (0.18%) = Cu (0.18%), which was evaluated by the sum of the extractable and carbonate-bound forms. The higher the bioavailability, the higher the ecological risk to the environment.

### 3.3. Pollution Assessment of the Heavy Metals 

#### 3.3.1. Contamination Factor and Pollution Load Index

The $C_{f}^{i}$ and *PLI* were calculated to evaluate the level of heavy metal pollution in the sediments (Table 5). The $C_{f}^{i}$ value and corresponding pollution level were classified into three classes: uncontaminated to moderately contaminated (1 ≤ $C_{f}^{i}$ < 3), moderately to heavily contaminated (3 ≤ $C_{f}^{i}$ < 6), and extremely contaminated ($C_{f}^{i}$ ≥ 6) [45]. The results showed that the average value of the $C_{f}^{i}$ decreased in the order of Hg > Zn > Cd > As > Pb > Cu > Cr > Ni. The corresponding pollution level suggested that, except for element Ni, all the detected heavy metals were above the polluted level, among which Hg was extremely contaminated. The above results indicated that Hg was the major pollutant, followed by Pb and Cd. 

The *PLI* provides an integrated assessment for all the determined elements, where *PLI* = 1 indicates that the concentration is at the same level as the background value, and a *PLI* value greater than 1 indicates contamination of the sediments. The results of the *PLI* values for the 12 sample sites showed deterioration of the sediment quality for all the sample points (*PLI* > 1). Sites S3, S4, S5, and S6 were all suspected to be moderately polluted (1 < *PLI* < 2), while the other sample sites were all confirmed to be heavily contaminated (*PLI* > 2). Overall, the pollution of the sediment in the northern part was more severe than that in the southern part.

#### 3.3.2. Potential Ecological Risk Index

The ecological risk assessment of the toxic metal contamination in the sediments of the inflow rivers was conducted using the *PERI*. The results of contamination degree are listed in Table 6. The grades and ranges of the different indexes are listed in Table S3. The average potential ecological risk of the 12 sampling sites for the different heavy metal elements when ranked from high to low was Hg > Cd > As > Pb > Cu > Zn > Cr > Ni. The contamination degree for the potential risk index ($E_{r}^{i}$) for As, Pb, Cu, Zn, Cr, and Ni was low risk; for Cd, it was high risk; and for Hg, it was extremely high risk. The *PERI* also showed that the ecological risk posed by heavy metals was much higher in the northern part. 

#### 3.3.3. Comparison of the Results from Different Methods

For the contamination degree of different heavy metals, the order calculated by $C_{f}^{i}\;$ was highly consistent with the results of $E_{r}^{i}$, except for element Cu, indicating that Hg, Cd, As, Pb were largely accumulated. This was probably associated with the large heavy metal emissions that were derived from human activities around Dianchi Lake, especially in the northern part. Research has shown that the lake has been affected by the release of heavy metals since the 1960s, and several studies have reported a high potential ecological risk of Hg, Cd, As, and Pb [34,50]. 

For the spatial distribution of risk, the *PLI* and *PERI* indexes both illustrated that the pollution risk in the densely populated and industrial-intensive northern part was higher than in the southern part, which reflected the close relationship between risk tendency and human activities. 

The *PLI* was evaluated from the perspective of enrichment effect, which is affected by the selection and determination of background values, while the *PERI* method is based on the toxicity coefficient and water sensitivity parameter, which could better reflect the pollution impact on the environment. Nevertheless, the coefficient and classification standards of the *PERI* were obtained by the study of eight pollutants (PCB, Hg, Cd, As, Pb, Cu, Zn, Cr), and should be adjusted according to the types and quantities of pollutants participating in the evaluation in the specific application. The adjustment schemes have great arbitrariness and are not uniform with each other, which also affects the reliability of the evaluation results. The metal fractions, especially for the available form, could better reflect the impact of toxic elements on the ecological environment; however, the assessment methods still need to be established. 

Overall, the above methods all produced reasonable evaluation results in the risk assessment of different metals and comprehensive evaluation of the study area, but all have their shortcomings. Thus, the importance of research of guidelines based on the speciation and bioavailability of sediment pollutants for establishing environmental standards and risk assessment methods was revealed. 

### 3.4. Source Identification of the Heavy Metals

**Figure 3:**
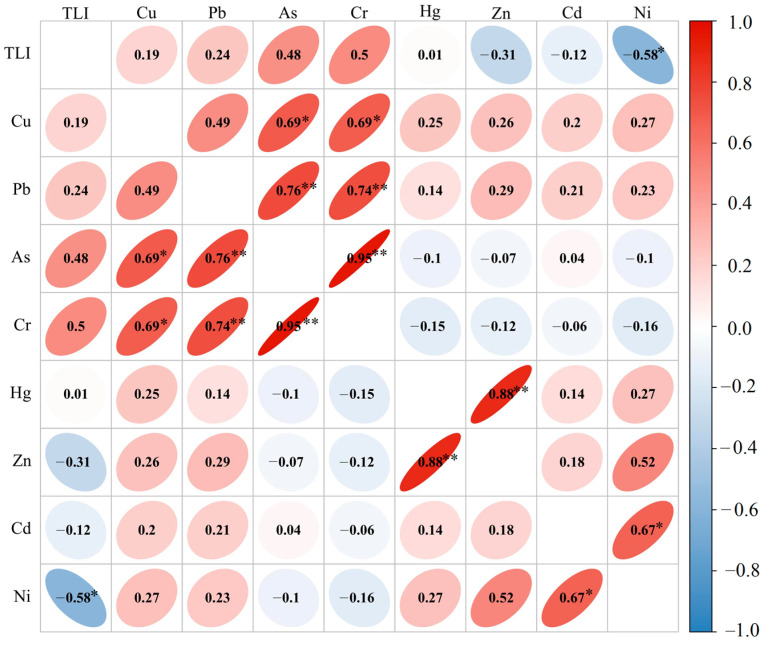
Correlation analysis for heavy metals in sediment (** *p* ≤ 0.01, * *p* ≤ 0.05).

The homology of the sediment metals at the sampling sites was evaluated using Pearson CA (Figure 3). The results showed strong significant positive correlations among As, Pb, and Cr (*r* > 0.7); significant positive correlations among As, Cu, and Cr (*r* > 0.6); and a significant negative correlation between Ni and *TLI*_∑_ (*r* < −0.6). It can be concluded that some heavy metals in the sediments were derived from a common source, particularly As, Cu, and Cr. Although the similarity between the sources of the different heavy metals could be speculated based on the CA, the contributions of the different sources are still undefined. Thus, the PCA method was adopted to conduct further analysis.

In this study, nine variables, that is, eight heavy metals and the *TLI*_∑_, were summarized using three principal components (Table 7), and they explained 68.31% of the total variance. The Kaiser–Meyer–Olkin (KMO) test and Bartlett’s test of sphericity were performed before conducting the PCA. The KMO value was >0.5, and the significance value of Bartlett’s test was <0.05, which indicated that the data were acceptable for the PCA [55,56]. The first principal component (PC1) explained 31.2% of the total variance, while the heavy metals Zn, Hg, Cu, and Pb had higher values for PC1, which indicated fossil fuel-generated pollution, probably from transportation and the chemical industry [57]. Then, PC2 explained 23.9% of the total variance, and the values for Cr, As, and *TLI*_∑_ were higher on PC2, which reflected the influence of the nutrients and their interaction with metals, which was mainly derived from fertilizer application due to agricultural activities. Additionally, PC3 explained 13.2% of the total variance, and the values of Cd and Ni were higher on PC3 (for an illustration of eigenvalues for PCA, see Figure S1). Although Cd is used in industries such as ceramics, electroplating, and pigments, it was reported to be more related to nonferrous mining and metal-handling activities [6]. The results were in accordance with the industrial distribution of Kunming, where agriculture and chemical industry account for a considerable proportion of the urban economy and could provide a data basis for policy setting in the pollution management of the Dianchi Lake Basin. Nevertheless, larger-scale and higher-frequency investigation is still needed for more precise management.

## 4. Conclusions 

The results of this study indicate that the heavy metal accumulation in the sediment of the Dianchi Lake Basin is a serious environmental problem. The results of different assessment methods using the risk indexes of the *C_f_*, *PLI*, and *PERI* were largely consistent, with most of the heavy metals being at or above significant pollution levels, especially the metal elements Hg and Cd, and the spatial characteristic of pollution risk was in accordance with the population density and industrial intensity. 

In the chemical speciation analysis, the distribution characteristics were distinct for different metal elements. Nevertheless, the bioavailability decreased in the order of Cd > Zn > Hg > Pb > As > Cr = Cu, and most of the metals occurred in nonbioavailable forms, such as the Fe/Mn oxide-bound form, the organic matter-bound form, and residual fractions. 

The Pearson CA indicated that there was a significant correlation between As, Pb, Cu, and Cr, which reflected an industrial chemical pollution source. The PCA results showed that there were three principal components of the heavy metal pollution in the sediment, which were likely derived from transportation (31.2%), the chemical industry (23.9%), and agricultural pollution sources (13.2%). 

The findings suggest that industrial and transportation pollution should be prioritized for the management of the Dianchi Lake Basin, especially for Hg, Zn, Cd, As, Pb, and Cr. Additionally, more research is needed to evaluate the potential environmental risks and their correlation with the chemical fractions of heavy metals in order to achieve certain improvements in the health and risk evaluation methodology for urban lakes.

## Figures and Tables

**Table 1:** Statistical results of surface water quality.

Item	Chl-a	COD_Mn_	TN	TP	As	*TLI*_∑_
	μg/L	mg/L	mg/L	mg/L	μg/L	
Mean	32.6	4.41	3.23	0.136	1.28	43.3
Median	11.0	3.70	2.11	0.135	1.10	44.3
CV%	118%	39.6%	73.1%	49.1%	77.5%	21.2%
Std.	38.4	1.75	2.36	0.067	0.994	9.17
Min	--	2.90	1.02	0.030	--	25.1
Max	128	9.70	7.58	0.290	5.00	58.6
Skewness	1.228	1.596	0.832	0.531	2.342	−0.448
Kurtosis	0.557	2.69	−0.907	0.104	7.992	−0.676

**Table 2:** The variance of seasonal differences in water quality of surface water.

Item	Unit	Average		Variance		MS	F	*p*-Value
		Wet	Dry	Wet	Dry			
Chl-a	μg/L	32.7	32.5	1539	1540	0	0.00	0.9918
COD_Mn_ *	mg/L	5.50	3.33	3.70	0.11	28.38	14.92	0.0008
TN *	mg/L	5.11	1.38	3.99	0.029	72.84	36.23	6.94 × 10^−6^
TP	mg/L	0.13	0.14	0.00	0.00	0.00	0.20	0.6574
As *	μg/L	1.82	0.75	1.20	0.25	6.83	9.44	0.0056
*TLI*_∑_		46.7	39.95	76.41	74.40	273.92	3.63	0.0698

“*” refers to significant variability.

**Table 3:** Statistical results of heavy metal content in inflow river of Dianchi Lake sediments (mg/kg).

Name	Hg	Pb	As	Cd	Cu	Zn	Ni	Cr
Mean	0.71	63.07	21.94	1.41	104	307	6.06	114
Median	0.29	62.95	17.80	0.20	99.50	212	0	108
CV%	113%	43.1%	83.70%	200%	49.90%	68.30%	244%	33.90%
Std.	0.81	27.20	18.35	2.82	51.89	210	14.81	38.51
Min	0.06	25.80	6.70	0	25.80	61.70	0	61.00
Max	3.29	122	96.60	11.10	227	689	48.90	220
Skewness	1.77	0.22	3.16	2.85	0.50	0.69	2.37	1.10
Kurtosis	3.30	−0.94	12.21	7.76	−0.32	−1.22	4.18	1.29
Background value	0.06	34.20	5.50	0.35	62.00	95.00	42.40	93.00

**Table 4:** Seasonal variance of heavy metal content in inflow river of Dianchi Lake sediments.

Element	Average (mg/kg)		Variance		MS	F	*p*-Value
	Wet	Dry	Wet	Dry			
As	27.40	16.48	523.98	115.29	716	2.2405	0.1487
Hg	0.72	0.70	0.48	0.88	0.0013	0.0020	0.9650
Cu	101.78	106.20	1776	3845	117	0.0416	0.8402
Pb	68.63	57.51	491.56	985.75	741	1.0038	0.3273
Zn	326.33	288.48	40,036	51,428	8600	0.1880	0.6688
Cd	1.03	1.78	6.96	9.33	3.3750	0.4144	0.5264
Cr	109.27	109.05	635	2160	0.2841	0.0002	0.9888
Ni	10.95	1.17	396.29	10.12	574	2.8271	0.1068

**Table 5:** Statistical summary of the $C_{f}^{i}$ and *PLI* of heavy metal contamination for the study site.

Site	*CF*_i_								*PLI*
	As	Hg	Cu	Pb	Zn	Cd	Cr	Ni	
S1	9.95	3.22	2.10	1.85	2.09	0.59	1.76	0.00	2.31
S2	4.16	3.03	1.06	0.95	1.73	6.33	1.04	0.00	2.28
S3	1.88	2.38	2.81	0.88	1.90	0.07	1.33	0.00	1.07
S4	2.66	2.29	0.77	1.62	2.02	2.73	0.80	0.00	1.87
S5	6.26	3.41	1.05	2.75	3.66	0.14	2.11	0.00	1.78
S6	2.40	1.97	0.53	2.26	3.85	0.97	0.94	0.00	1.66
S7	2.45	11.60	1.24	1.33	5.95	0.36	1.00	0.00	2.15
S8	4.61	37.27	2.35	2.45	9.69	4.00	1.30	0.00	5.81
S9	1.98	19.18	1.35	1.17	5.71	0.00	0.94	0.00	3.21
S10	2.76	19.45	2.27	1.95	9.92	2.61	0.97	0.23	4.29
S11	3.24	14.65	1.97	2.25	6.31	1.50	1.16	0.22	3.55
S12	5.50	14.70	2.63	2.66	6.66	28.96	1.33	0.32	6.91
Average	3.99	11.10	1.68	1.84	4.96	4.02	1.22	0.06	3.07

**Table 6:** Statistical summary of the $E_{r}^{i}$ and *PERI* of heavy metal contamination for the study site.

Site	$E_{r}^{i}$								*PERI*
	Hg	Pb	As	Cd	Zn	Ni	Cu	Cr	
S1	128.8	9.3	99.5	17.6	2.1	0.0	10.5	3.5	271.1
S2	121.3	4.7	41.6	189.9	1.7	0.0	5.3	2.1	366.6
S3	95.3	4.4	18.8	2.1	1.9	0.0	14.0	2.7	139.3
S4	91.6	8.1	26.6	81.9	2.0	0.0	3.9	1.6	215.7
S5	136.6	13.7	62.6	4.3	3.7	0.0	5.3	4.2	230.4
S6	78.8	11.3	24.0	29.1	3.8	0.0	2.6	1.9	151.5
S7	464.1	6.7	24.5	10.7	6.0	0.0	6.2	2.0	520.1
S8	1490.6	12.3	46.1	120.0	9.7	0.0	11.7	2.6	1693.0
S9	767.2	5.8	19.8	0.0	5.7	0.0	6.7	1.9	807.2
S10	778.1	9.8	27.6	78.4	9.9	0.5	11.4	1.9	917.6
S11	585.9	11.3	32.4	45.0	6.3	0.4	9.8	2.3	693.4
S12	587.8	13.3	55.0	868.7	6.7	0.6	13.2	2.7	1547.9
Average	443.8	9.2	39.9	120.6	5.0	0.1	8.4	2.4	629.5

**Table 7:** Results of principal component analysis (PCA).

Component	PC1	PC2	PC3
Eigenvalue	2.805	2.151	1.191
% of variance	31.172	23.900	13.238
Cumulative %	31.172	55.072	68.310
Hg	0.456	−0.080	−0.200
Pb	0.160	0.265	0.100
As	−0.059	0.384	0.089
Cd	−0.136	0.066	0.547
Cu	0.209	0.089	0.095
Zn	0.437	−0.056	−1.00
Ni	−0.084	−0.059	0.511
Cr	0.005	0.372	−0.039
*TLI_∑_*	−0.023	0.286	−0.247

## Data Availability

The raw data supporting the conclusions of this article will be made available by the authors on request.

## Supplementary Materials

The following supporting information can be downloaded at: https://www.mdpi.com/article/10.3390/toxics12050322/s1, Figure S1: Eigenvalues for principal component analysis (PCA); Table S1: Values of $r_{ij}$ and $r_{ij}^{2}$ of lakes (reservoir) in China; Table S2: Grading value for nutrient status; Table S3: Grades and ranges of $E_{r}^{i}$ and *PERI*.
