# Supplementary material for: Pollution Characteristics and Risk Assessment of Heavy Metals in the Sediments of the Inflow Rivers of Dianchi Lake, China
Source: Toxics. 2024 Apr 29;12(5):322. doi: 10.3390/toxics12050322 (PMC11125836; doi:10.3390/toxics12050322)
Supplement: Supplementary file 1 [file toxics-12-00322-s001.zip › toxics-2978476-supplementary.pdf]

Table S1. Values of r_{ij} and r_{ij}^2 of lakes (reservoir) in China

Parameter	chl-a	TP	TN	SD	COD_{Mn}
r_{ij}	1	0.84	0.82	-083	0.83
r_{ij}^2	1	0.7056	0.6724	0.6889	0.6889

Calculation formula for nutrient status index:

$$TLI(chl\text{-}a) = 10\times(2.5 + 1.086\ln chla) \tag{1}$$

$$TLI(TP) = 10\times(9.436 + 1.624\ln TP) \tag{2}$$

$$TLI(TN) = 10\times(5.453 + 1.694\ln TN) \tag{3}$$

$$TLI(SD) = 10\times(5.118 - 1.94\ln SD) \tag{4}$$

$$TLI(COD_{Mn}) = 10\times(0.109 + 2.661\ \ln COD_{Mn}) \tag{5}$$

In the Eq. (1) to Eq. (5), the unit of chl-a was $mg\cdot m^{-3}$, the unit of SD was m, the unit of TP, TN and COD_{Mn} were $mg\cdot L^{-1}$.

Table S2. Grading value for nutrient status

Value of TLI_{Σ}	Grading
$TLI_{\Sigma}<30$	Oligotrophe
$30\leqslant TLI_{\Sigma}\leqslant 50$	Mesotropher
$TLI_{\Sigma}>50$	Eutropher
$50<TLI_{\Sigma}\leqslant 60$	Light Eutropher
$60<TLI_{\Sigma}\leqslant 70$	Middle Eutropher
$TLI_{\Sigma}>70$	Hyper Eutropher

Table S3. Grades and ranges of E_r^i and *PERI* (Hakanson, 1980)

E_r^i	Grades of E_r^i for metal *i*	*PERI*	Grades of *PERI*
$E_r^i < 40$	Low risk	$PERI < 150$	Low risk
$40\leq E_r^i < 80$	Moderate risk	$150\leq PERI < 300$	Moderate risk
$80\leq E_r^i < 160$	High risk	$300\leq PERI < 600$	High risk
$160\leq E_r^i < 320$	Very high risk	$PERI \geq 600$	Very high risk
$E_r^i \geq 320$	Extremely high risk		

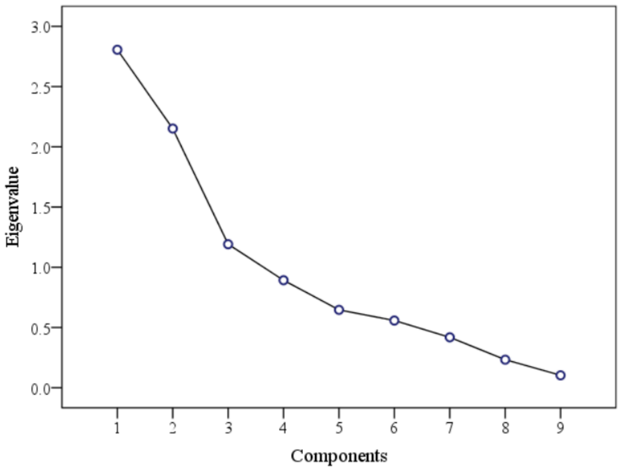

Figure S1. Eigenvalues for principal component analysis (PCA).
